# Genetic Diversity and Population Structure of *Vibrio parahaemolyticus* Isolated From Clinical and Food Sources

**DOI:** 10.3389/fmicb.2021.708795

**Published:** 2021-07-27

**Authors:** Min He, Tao Lei, Fufeng Jiang, Jumei Zhang, Haiyan Zeng, Juan Wang, Moutong Chen, Liang Xue, Shi Wu, Qinghua Ye, Rui Pang, Yu Ding, Qingping Wu

**Affiliations:** ^1^School of Bioscience and Bioengineering, South China University of Technology, Guangzhou, China; ^2^Guangdong Provincial Key Laboratory of Microbial Safety and Health, State Key Laboratory of Applied Microbiology Southern China, Institute of Microbiology, Guangdong Academy of Sciences, Guangzhou, China; ^3^School of Food and Biological Engineering, Shaanxi University of Science and Technology, Xi’an, China

**Keywords:** *Vibrio parahaemolyticus*, genetic diversity, population structure, clinical isolates, food isolates.

## Abstract

*Vibrio parahaemolyticus* is a common foodborne pathogen that causes gastroenteritis worldwide. Determining its prevalence and genetic diversity will minimize the risk of infection and the associated economic burden. Multilocus sequence typing (MLST) is an important tool for molecular epidemiology and population genetic studies of bacteria. Here, we analyzed the genetic and evolutionary relationships of 162 *V. parahaemolyticus* strains isolated in the Guangdong Province, China, using MLST. In the study, 120 strains were isolated from food samples, and 42 strains were isolated from clinical samples. All strains were categorized into 100 sequence types (STs), of which 58 were novel (48 from the food isolates and 10 from the clinical isolates). ST415 was the most prevalent ST among the food isolates, while ST3 was the most prevalent ST among the clinical isolates. Further, 12 clonal complexes, 14 doublets, and 73 singletons were identified in all ST clusters, indicating high genetic diversity of the analyzed strains. At the concatenated sequence level, non-synonymous sites in both, food and clinical isolates, were associated with purifying selection. Of note, the *dN*/*dS* ration was greater than 1 for some housekeeping genes in all isolates. This is the first time that some loci under positive selection were identified. These observations confirm frequent recombination events in *V. parahaemolyticus*. Recombination was much more important than mutation for genetic heterogeneity of the food isolates, but the probabilities of recombination and mutations were almost equal for the clinical isolates. Based on the phylogenetic analysis, the clinical isolates were concentrated in the maximum-likelihood tree, while the food isolates were heterogeneously distributed. In conclusion, the food and clinical isolates of *V. parahaemolyticus* from the Guangdong Province are similar, but show different evolutionary trends. This may help prevent large-scale spread of highly virulent strains and provides a genetic basis for the discovery of microevolutionary relationships in *V. parahaemolyticus* populations.

## Introduction

*Vibrio parahaemolyticus*, a halophilic, Gram-negative bacterium, is commonly found in estuarine and marine environments worldwide. This bacterium is an important pathogen of human. It typically causes acute gastroenteritis, wound infections, and septicemia, with high contamination rates worldwide (Ralph and Currie, 2007; Zhang and Orth, 2013). It is also considered one of the most prevalent foodborne pathogens (Letchumanan et al., 2014).

The main virulence factors of *V. parahaemolyticus* are thermostable direct hemolysin (TDH), TDH-related hemolysin (TRH), thermolabile hemolysin (TLH), three type 3 secretion systems (T3SS1, T3SS2α, and T3SS2β) and two type 6 secretion systems (T6SS1, T6SS2) (Park et al., 2004; Zhang and Austin, 2005; Okada et al., 2009; Hiyoshi et al., 2010). TDH acts directly on red blood cells and shows hemolytic and enterotoxin activity, cardiotoxicity, and cytotoxicity (Matsuda et al., 2009; Hiyoshi et al., 2010). TRH is composed of 189 amino acids and shares 54.8–68.8% sequence identity with TDH. It is present in a small number of clinical isolates and is encoded by the *trh1* and *trh2* genes (Kishishita et al., 1992). TLH is an atypical phospholipase that causes hemolysis only in the presence of lecithin (Zhang and Austin, 2005). T3SS1 mainly affects biofilm formation, movement, and cytotoxicity and contributes to the survival of *V. parahaemolyticus* in the environment. T3SS2 is involved in the negative regulation of cellular inflammatory response, which helps the bacterium to evade the immune response of the host (Calder et al., 2014). Finally, T6SS1 enhances the environmental adaptability of *V. parahaemolyticus* in the marine environment, while T6SS2 allows *V. parahaemolyticus* to adhere to host cells (Yu et al., 2012).

Many *V. parahaemolyticus* serotypes are associated with infection. For instance, the pandemic serotype O3: K6 was the main cause of a global outbreak of *V. parahaemolyticus* infection (Nair et al., 2007). In fact, many countries have reported outbreaks of *V. parahaemolyticus* infection, including the US (Shaw et al., 2015), Italy (Caburlotto et al., 2016), Brazil (Leal et al., 2008), Japan (Arakawa et al., 1999), and Bangladesh (Bhuiyan et al., 2002). Consumption of raw or undercooked seafood contaminated with *V. parahaemolyticus* is the main cause of outbreaks of *V. parahaemolyticus* infection (McLaughlin et al., 2005; Pal and Das, 2010). In China, where high amounts of seafood are consumed, most foodborne outbreaks and cases of infectious diarrhea are caused by *V. parahaemolyticus*, especially in the coastal areas (Liu et al., 2018; Paudyal et al., 2018). Further, clinical isolates in China usually represent the pandemic serotype O3: K6 and several of its serovariants (e.g., O1: KUT, O1: K56, and O4: K68) (Yu et al., 2011; Li et al., 2014).

The risk of *V. parahaemolyticus* infection and the associated economic burden can be minimized by determining the prevalence and genetic diversity of this pathogen at the population level. Molecular biology methods have rapidly advanced in recent years, and many molecular typing techniques are used for molecular epidemiological studies of *V. parahaemolyticus* (Gonzalez-Escalona et al., 2008; Chen et al., 2012). Multilocus sequence typing (MLST) of *V. parahaemolyticus* isolates, developed by Gonzalez-Escalona et al. (2008), is an important tool for molecular epidemiology and population genetic studies of this bacterium (Urwin and Maiden, 2003; Turner and Feil, 2007; Gonzalez-Escalona et al., 2008). However, although a number of studies have used MLST to investigate the genetic diversity of *V. parahaemolyticus* in China, these studies either focused on clinical isolates or food isolates. A comparative analysis of food and clinical isolates is missing.

In the current study, we performed genetic and population analysis of 162 food and clinical isolates of *V. parahaemolyticus* from the Guangdong Province, China. We compared their genetic diversity, prevalence, and population structure using MLST and investigated possible evolutionary relationships of these isolates by sequence polymorphism analysis. We also determined the differences in the genetic population structure of foodborne and clinical *V. parahaemolyticus* isolates. The findings provide genetic evidence on the microevolutionary relationships among *V. parahaemolyticus* isolates from different sources, with the potential to support the development of new strategies for the prevention of infection.

## Materials and Methods

### Bacterial Isolates

In the study, 162 *V. parahaemolyticus* isolates, including 120 food isolates and 42 clinical isolates, from the Guangdong Province, China, were analyzed (Supplementary Table 1). All isolates were stored and maintained in the authors’ laboratory. Aquatic product and ready-to-eat foods samples were collected in Guangdong province, China, from January to June in 2016. After collection, all samples were placed in sterile sealed plastic bags, transported to the laboratory in a cold box and analyzed immediately. According to National Standards of the People’s Republic of China (GB 4789.7-2013), *V. parahaemolyticus* were isolated and identified. In brief, each sample (25.0 g) was homogenized in a stomacher bag with 225 mL alkaline peptone water containing 3% (w/v) NaCl. Homogenates were incubated at 37°C for 16–18 h. Samples were streaked onto thiosulfate citrate bile-salt sucrose (TCBS) agar plates and incubated at 37°C for 18–24 h. Presumptive colonies (green or blue green colonies, 2–3 mm in diameter) were streaked onto Chromogenic *Vibrio* Medium and incubated at 37°C for 24 h. Mauve colonies were selected for further *V. parahaemolyticus* identification by analysis of oxidase activity, Gram staining, 3.5% NaCl triple sugar iron (TSI) tests, halophilism tests, and API 20E diagnostic strips (Biomerieux Company, France). Thirty-one clinical isolates collected from patients in Guangdong, China were gifted by the Nanshan, Shenzhen Center for Disease Control and Prevention. Eleven clinical isolates were isolated in the authors’ laboratory.

### Polymerase Chain Reaction Amplification and Sequencing

Before the analysis, the bacteria were grown in tryptic soy broth (HuanKai Microbial, Guangzhou, China) supplemented with 3% (w/v) NaCl at 37°C overnight. Genomic DNA was extracted from bacteria using a Genomic DNA Extraction kit (Magen Biotech, Guangzhou, China), according to the manufacturer’s instructions. Seven loci (housekeeping genes) were selected for MLST analysis, based on a previously published MLST scheme for *V. parahaemolyticus* (Gonzalez-Escaloa et al., 2017). These were: *recA*, encoding RecA protein; *dnaE*, encoding alpha subunit of DNA polymerase III; *gyrB*, encoding B subunit of DNA gyrase; *dtdS*, encoding threonine dehydrogenase; *pntA*, encoding alpha subunit of transhydrogenase; *pyrC*, encoding dihydroorotase; and *tnaA*, encoding tryptophanase. Among them, *recA*, *dnaE*, and *gyrB* are located on chromosome I, and *dtdS*, *pntA*, *pyrC*, and *tnaA* are located on chromosome II. The genes were amplified following the polymerase chain reaction protocol available on the *V. parahaemolyticus* PubMLST website^1^ and using the primer sets listed in Supplementary Table 2. The products of polymerase chain reaction were then subjected to 1.5% (w/v) agarose gel electrophoresis with gold-view staining to purify, and bi-directionally sequenced using an ABI-3700XL instrument (BGI Instruments, Guangdong, China). The raw sequencing data of all strains were in Supplementary Data Sheet 1–8.

### MLST Analysis

For MLST analysis, AB1 sequencing files were first imported into Bionumerics v. 7.6 (Applied Maths, Sint-Martens-Latem, Belgium). The sequences were edited and complementary locus fragments from individual isolates were assembled, trimmed and aligned by Bionumerics v. 7.6. Next the allele name and sequence type (ST) of each isolate were determined by querying the gene sequence in the PubMLST database. Whenever novel alleles were identified, their sequences and new allelic profiles were submitted to the PubMLST database, and new numerical identifiers were assigned.

Global optimal eBURST (goeBURST) (Francisco et al., 2009) analysis of related STs was performed using the Phyloviz software,^2^ and the clonal relationships among isolates were obtained. A single clonal complex (CC) was defined as a complex in which at least six of the seven alleles analyzed were identical among the isolates. Single- and double-locus variants (SLVs and DLVs, respectively) were defined as two STs differing at a single locus or at two loci, respectively. The goeBURST algorithm was also used to take a population snapshot of groups sharing between 0 and 7 loci, to create a full MLST map, where all STs are connected (Francisco et al., 2009).

### Phylogenetic Analysis

MUSCLE (Edgar, 2004) was used to perform multiple sequence alignments of each locus. A majority consensus phylogeny of the concatenated loci (3,682 bp) was constructed using the maximum likelihood (ML) method implemented in MEGA X (Kumar et al., 2018). The nodes in the ML tree were evaluated by 1000 bootstrap-based re-sampling.

### Population Genetics Analysis

The in-frame sequences were concatenated in the order *dnaE-gyrB-recA-dtdS-pntA-pyrC-tnaA* to produce a cumulative sequence (3682 bp in length) for each strain. DNASP v. 6 was used to calculate the number of polymorphic sites, nucleotide diversity (π), and the *dN*/*dS* ratio for individual loci and the concatenated sequences, to evaluate locus variation in the selected isolates (Rozas et al., 2017). *Pi* (π) represents the average number of nucleotide differences at each site between two randomly selected strains. The higher the π value, the higher the gene nucleotide polymorphism. The *dN*/*dS* ratio is used for the selection and hypothesis tests for neutrality (*dN* = *dS*): non-synonymous sites are considered to be under purifying selection (negative selection) if *dN*/*dS* < 1; *dN*/*dS* > 1 indicates positive selection; and *dN*/*dS* = 1 indicates neutrality. In the analysis, *dN* represents the number of synonymous substitutions per synonymous locus, and *dS* represents the number of non-synonymous substitutions per non-synonymous locus. Further, recombinant *phi* test of individual loci in the whole strain collection was performed using SplitsTree v. 4.0 (Huson and Bryant, 2006). For the calculated *p*-values, *p* < 0.05 indicated the occurrence of recombination.

The standardized index of association (*I_*A*_^*S*^*) was calculated using LIAN v. 3.7 (Haubold and Hudson, 2000) to assess the population structure of *V. parahaemolyticus*; 10,000 iterations in the Monte Carlo mode were used. For all pairwise comparisons of allelic profiles, *I_*A*_^*S*^* was defined as the observed variance of the distribution of allelic mismatches divided by the expected variance of the free recombination population minus 1. Statistics were used to verify the null hypothesis of linkage equilibrium: if alleles are in a linkage equilibrium (i.e., independently distributed at all loci analyzed), recombination occurs frequently and *I_*A*_^*S*^* = 0.

LDhat program in RDP4 package (Martin et al., 2017) was used to calculate the mean per-site recombination (*rho*)/mutation (*theta*) ratio of concatenated sequences of STs with 1,000,000 MCMC updates. The ratio indicates the probability that a given site is altered by recombination and mutation. Hence, the *rho*/*theta* ratio is used to measure the effect of recombination on the diversity of sample relative to a given mutation.

## Results

### *V. parahaemolyticus* Isolates From Clinical and Food Sources Show High Levels of Nucleotide and Allelic Diversity

MLST analysis revealed 98 unique STs, 58 (59.18%) of which were novel, as compared with the previous PubMLST database entries. Among the novel STs, 48 (57.14%) were identified among the food isolates and 10 (66.67%) were identified among the clinical isolates (Table 1). Further, 77 unique STs were represented by single isolates, and 21 STs were associated with more than one strain. ST3 was the most frequently detected ST, represented by 23 clinical isolates. ST1820 contained both food and clinical isolates. The observed number of alleles for each locus ranged from 47 (*tnaA*) to 70 (*gyrB*). Interestingly, *dnaE* was the locus showing the most new alleles (*n* = 10). Accordingly, the nucleotide and allelic diversity of the seven loci were significantly different (Table 2). The nucleotide diversity ranged from 0.00979 to 0.03812 for all isolates, with the greatest diversity observed for *tnaA* (0.03812). However, *pntA* was the least polymorphic locus, with the lowest nucleotide diversity per site (π = 0.00979). With regard to the source of the isolates, the values ranged from 0.00604 (*dnaE*) to 0.0178 (*dtdS*) for the clinical isolates and from 0.00852 (*pntA*) to 0.04687(*tnaA*) for the food isolates (Tables 3, 4). The food isolates showed higher nucleotide diversity than the clinical isolates. Collectively, both, the food and clinical isolates showed high levels of nucleotide and allelic diversity at all loci studied.

**TABLE 1:** Properties of each locus and sequence type.

Regions	Number of		Number of new			
	alleles/STs		alleles/STs		Whole	
			
	Clinical isolates	Food isolates	Clinical isolates	Food isolates	Total	New
*dnaE*	10	53	0	10	57	10
*gyrB*	10	64	2	7	70	9
*recA*	12	52	0	5	57	5
*dtdS*	12	59	0	5	68	5
*pntA*	11	45	1	0	52	1
*pyrC*	11	55	0	2	59	2
*tnaA*	10	43	1	2	47	3
STs	15	84	10	48	98	58

**TABLE 2:** Nucleotide and allelic sequences diversity of all isolates.

Chromosome	Locus	Fragment length (bp)	Number of alleles/STs	Number of new alleles/STs	Number of polymorphic sites	Nucleotide diversity (π)	*dS*	*dN*	*dN*/*dS*
Chromosome I	*dnaE*	557	57	10	54	0.01042	0.02443	0.00558	0.22841
	*gyrB*	592	70	9	47	0.01362	0.00129	0.01715	13.29457
	*recA*	729	57	5	62	0.02249	0.00373	0.02778	7.44772
Chromosome II	*dtdS*	458	68	5	58	0.02226	0.00384	0.02907	7.57031
	*pntA*	430	52	1	38	0.00979	0.03896	0.00075	0.01925
	*pyrC*	493	59	2	45	0.01000	0.00533	0.01157	2.17073
	*tnaA*	423	47	3	388	0.03812	0.07168	0.02820	0.39342
	Concatenated	3682	98	58	692	0.01787	0.02185	0.01676	0.76705

**TABLE 3:** Nucleotide and allelic sequences diversity of clinical isolates.

Chromosome	Locus	Fragment length (bp)	Number of alleles/STs	Number of new alleles/STs	Number of polymorphic sites	Nucleotide diversity (π)	*dS*	*dN*	*dN*/*dS*
Chromosome I	*dnaE*	557	10	0	14	0.00604	0.01776	0.00177	0.09966
	*gyrB*	592	10	2	20	0.00943	0.00000	0.01135	0.00000
	*recA*	729	12	0	44	0.01275	0.00354	0.01489	4.20621
Chromosome II	*dtdS*	458	12	0	28	0.01780	0.00146	0.02589	17.73288
	*pntA*	430	11	1	15	0.00841	0.03617	0.00029	0.00802
	*pyrC*	493	11	0	19	0.00731	0.00230	0.01001	4.35217
	*tnaA*	423	10	1	16	0.00715	0.02835	0.00015	0.00529
	Concatenated	3682	15	10	156	0.00998	0.01436	0.00875	0.60933

**TABLE 4:** Nucleotide and allelic sequences diversity of food isolates.

Chromosome	Locus	Fragment length (bp)	Number of alleles/STs	Number of new alleles/STs	Number of polymorphic sites	Nucleotide diversity (π)	*dS*	*dN*	*dN*/*dS*
Chromosome I	*dnaE*	557	53	10	52	0.01141	0.02434	0.00675	0.27732
	*gyrB*	592	64	7	47	0.01393	0.00169	0.01742	10.30769
	*recA*	729	52	5	61	0.02069	0.00293	0.02611	8.91126
Chromosome II	*dtdS*	458	59	5	56	0.02142	0.00425	0.02738	6.44235
	*pntA*	430	45	0	35	0.00852	0.03320	0.00089	0.02681
	*pyrC*	493	55	2	43	0.00981	0.00621	0.01101	1.77295
	*tnaA*	423	43	2	388	0.04687	0.07717	0.03792	0.49138
	Concatenated	3682	84	48	682	0.01843	0.02258	0.01717	0.76041

### Evidence of Purifying and Positive Selection in *V. parahaemolyticus* Isolates From Clinical and Food Sources

The *dN*/*dS* ratios (Table 2) were < 1 for *dnaE*, *pntA*, and *tnaA* and > 1 for *gyrB*, *recA*, *dtdS*, and *pyrC*. A similar trend was observed in the food and clinical isolates, except for *gyrB*, in which no non-synonymous changes were identified in the clinical isolates (Tables 3, 4). To the best of our knowledge, this is the first study reporting *dN*/*dS* ratios > 1 for the four housekeeping genes. Since *dN*/*dS* < 1 indicates that the evolutionary rate of non-synonymous loci is lower than that of synonymous loci, the *dnaE*, *pntA*, and *tnaA* genes were mainly affected by purifying selection during evolution. However, *dN*/*dS* > 1 indicated positive selection for *gyrB*, *recA*, *dtdS*, and *pyrC*. Of note, the π-value and mean *dN*/*dS* ratio of food isolates (0.01843 and 0.76041, respectively) were 1.8- and 1.2-fold higher, respectively, than those of clinical isolates (0.00998 and 0.60933, respectively) (Tables 4, 3). Collectively, these observations suggest that the purifying selection of food and clinical isolates was similar overall, while the genetic evolution of food isolates was faster than that of clinical isolates.

### *V. parahaemolyticus* Isolates From Clinical and Food Sources Show High Level of Recombination

The *phi* test *p*-values for the concatenated sequences of all strains were < 0.001, indicating a significant evidence of recombination. This was also true for the clinical and food isolates considered separately, suggesting recombination in these strains. The *rho*/*theta* ratio for the concatenated sequences was 8.703, suggesting that recombination was approximately 8.7 times more likely to occur than point mutations during strain evolution. It is consistent with the previous study that *rho*/*theta* > 1, indicating that recombination had a more important impact on genetic heterogeneity and diversity than mutation (Dong et al., 2016). The *rho*/*theta* values for food and clinical isolates were 14.264 and 1.476, respectively. Both these values were above 1. These observations suggest that recombination occurred more frequently than point mutations in the food isolates, while the probability of recombination was close to that of point mutation in the clinical isolates. Next, the standardized index of association (*I^*S*^_*A*_*) was calculated (Table 5). The *I^*S*^_*A*_* value for the whole strain collection was 0.6269, significantly different from zero (*p* < 0.0001). Considering the food and clinical strains separately, the *I^*S*^_*A*_* values were 0.4429 and 0.8361, respectively, and were both significantly positive (*p* < 0.0001). Importantly, the *I^*S*^_*A*_* values were consistent with the notion of recombination in the *V. parahaemolyticus* population. Collectively, these data indicate a tendency of linkage disequilibrium between the alleles, which is consistent with the existing research results (Han et al., 2015; Li et al., 2018).

**TABLE 5:** Recombination test and estimation.

Population (*n*)	ST (New ST)	*Phi*	Recombination					Linkage disequilibrium	
				
			*Theta*/*site*	*Rho*/*site*	LB^*a*^ 95%	UP^*b*^ 95%	*Rho*/*theta*	*I ^*S*^_*A*_*	*p-*value
Whole	98 (58)	<0.001	1.841 × 10^–2^	1.602 × 10^–1^	1.070 × 10^–1^	2.788 × 10^–1^	8.703	0.6269	<0.0001
Clinical isolates	15 (10)	<0.001	1.067 × 10^–2^	1.575 × 10^–2^	1.173 × 10^–2^	2.116 × 10^–2^	1.476	0.8361	<0.0001
Food isolates	84 (48)	<0.001	1.890 × 10^–2^	2.695 × 10^–1^	1.617 × 10^–1^	4.723 × 10^–1^	14.264	0.4429	<0.0001

*^*a*^Lower Bound.*

*^*b*^Upper Bound.*

### Identification of CCs

**FIGURE 1:**
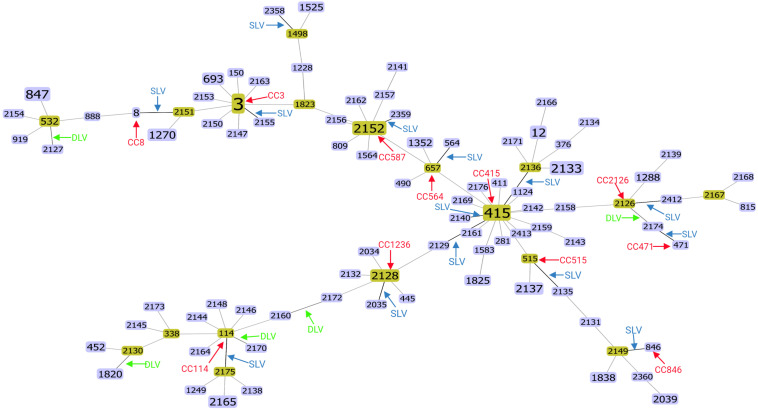
Full MLST map of *V. parahaemolyticus* isolates from food and clinical samples from Guangdong Province, based on nucleotide sequencing. Twelve clonal complexes (CC3, CC8, CC587, CC564, CC114, CC415, CC471, CC515, CC864, CC1236, CC2126, andCC1498), fourteen SLVs, five DLVs and 72 singletons were identified. All connections are shown. STs that are SLVs of each other are connected by black lines. STs that differ in two or more alleles are connected by light gray lines.

The goeBURST algorithm used in the current study resolved the 98 STs into twelve CCs (CC3, CC8, CC587, CC564, CC114, CC415, CC471, CC515, CC864, CC1236, CC2126, andCC1498) (Figure 1). Among them, except CC415 contains 4 STs, the remaining eleven CCs contain 2 STs. The clinical isolates contained three CCs, CC3, CC8, CC587, and the food isolates contained eight CCs, CC564, CC114, CC415, CC471, CC515, CC864, CC1236, CC2126, respectively. In addition, CC1498 contains both food and clinical isolates. Among the clinical isolates, CC3 was the largest CC, including 24 strains in 2 STs (ST3 and ST2155), of which ST3 contained 23 strains and ST2155 contained 1 strain. Among the food isolates, CC415 was the largest CC, including 11 strains of 4 STs (ST415, ST2129, ST2136, and ST2140), of which ST415 contained 8 strains, and the rest each contained 1 strain. While in food isolates, no ST3 was found in this study, and no STs belonging to the pandemic CC3 were found, which was consistent with the previous study that did not find ST3 and STs belonging to CC3 in food isolates (Dong et al., 2016). CC1498 contained 2 strains from 2 STs (ST1498 and ST2158), of which ST1498 contained one food isolate(*Vp266*) and ST2158 contained one clinical isolate(*r16*), indicating that *Vp266* and *r16* were genetically similar, *Vp266* may have potential pathogenicity, and *r16* may be derived from food contaminated by highly virulent strains. In addition, fourteen SLVs, five DLVs and 72 singletons were identified from all isolates (Figure 1). In conclusion, the food and clinical isolates contained different CC, suggesting significant evolutionary differences between the food and clinical strains.

### Phylogenetic Analysis

**FIGURE 2:**
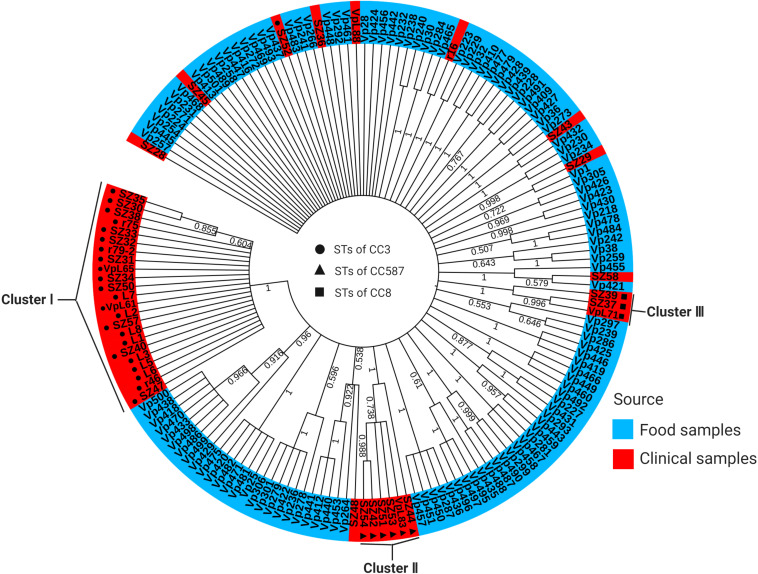
ML tree constructed using concatenated sequences of seven loci (*dnaE*-*gyrB*-*recA*-*dtdS*-*pntA*-*pyrC*-*tnaA*) of 162 *V. parahaemolyticus* isolates analyzed in the current study. Food isolates are denoted in blue, and clinical isolates are denoted in red. Circles, triangles, and squares with different shading represent the three CCs of clinical isolates identified by goeBURST. The scale represents the evolutionary distance. The numbers at the nodes represent bootstrap values based on 1000 replications.

ML tree based on the concatenated sequences of the seven housekeeping genes of 162 isolates is shown in Figure 2. Remarkably, the clinical isolates were concentrated in the tree, while the food isolates were heterogeneously distributed. This may be because the food samples were diverse. Specifically, the clinical isolates were separated into three major clusters (Figure 2). Cluster I (*n* = 23 isolates) was the most homogenous and was composed of one CC (CC3) and only one ST (ST3). Cluster II (*n* = 6 isolates) was composed of one CC (CC587) and two STs (ST2152 and ST2359). Cluster III (*n* = 3 isolates) was also composed of one CC (CC8) and two STs (ST8 and ST2151) (Figure 2 and Supplementary Table 1). However, the food isolates were grouped into many small clusters, each composed of different CCs and singletons; 31 small clusters were identified. Of note, a small number of clinical and food isolates appeared in the same cluster, and conversely, single clinical or food isolates formed separate one-isolate clusters.

## Discussion

In the current study, we analyzed the nucleotide diversity of *V. parahaemolyticus* isolates from food and clinical sources from Guangdong Province, China. We show that the isolates are highly diverse. Overall, 692 polymorphic sites and 100 alleles were identified by concatenated sequence analysis of 162 strains, with 58 novel STs, indicating a high level of genetic diversity of the MLST loci. Interestingly, the clinical isolates (66.67%) harbored a higher proportion of novel STs than the food isolates (55.81%). However, the food strains showed higher nucleotide diversity than the clinical isolates. Further, ST3 was the most common ST, in line with findings of a previous study analyzing samples from various countries (Han et al., 2014).

In the current study, the *dN*/*dS* ratio of the concatenated sequences was < 1. However, at the locus level, this was only true for three loci, while the remaining four loci studied showed *dN*/*dS* ratios > 1. This indicated that the three loci were subjected to purifying selection, while the remaining four were subjected to positive selection. To the best of our knowledge, this is the first study showing positive selection of housekeeping genes. There are two possible explanations for these findings. The first is related to environmental stressors, such as antibiotic therapy. Extensive antibiotic use is a contributing factor to the increasing incidence of antimicrobial-resistant strains. In fact, according to a recent report, some *V. parahaemolyticus* isolates are resistant to many commonly used antibiotics, including kanamycin, streptomycin, and ampicillin, because of excessive use of antibiotics in human and aquaculture systems (Park et al., 2018). Of note, all isolates analyzed in this study show various levels of antibiotic resistance (Xu et al., 2016; Xie et al., 2017, 2019). Therefore, to survive under the selective pressure, these *V. parahaemolyticus* strains had to adapt fast to the external environment, probably by acquiring non-synonymous mutations in some housekeeping genes (rather than acquiring synonymous mutations), in line with what has been reported by others (Yang, 2006). The second explanation relates to the adequateness of using the *dN*/*dS* ratio, highlighting a potential limitation of this study. Although this ratio is often calculated for sequences sampled from a single population, it was originally developed for the analysis of distantly divergent sequences. Consequently, this approach ignores the existence of polymorphisms and relies on the indirect assumption that new mutations are instantaneously fixed. This assumption is usually violated and is only reasonable for distantly related species. Of note, violation of the underlying assumption leads to a time-dependence of sequence divergence and biased estimates of the *dN*/*dS* ratio, especially for closely related species, where the contribution of ancestral and lineage-specific polymorphisms to sequence divergence is substantial (Mugal et al., 2020). In fact, it has been reported that when a single population under selection is sampled, the *dN*/*dS* ratio is relatively insensitive to the selection coefficient. Furthermore, for population samples, the relationship between selection and the *dN*/*dS* ratio does not follow a monotonic function; therefore, it may be impossible to infer selection pressures from the *dN*/*dS* ratio (Kryazhimskiy and Plotkin, 2008). All of the above point to a need to improve the *dN*/*dS* estimation for closely related species by traditional analysis methods. Importantly, the joint usage of polymorphism and divergence data may assist in the inference of selection of closely related species (Mugal et al., 2020). Regardless, high levels of variation generated by purifying and positive selection will ensure a good discriminatory power of MLST and facilitate the subsequent phylogenetic inference.

Various mechanisms of recombination operate in bacteria, such as transformation, phage transduction, and conjugation, which promote the replacement of DNA regions of core genes with homologous sequences from strains of the same or closely related species (Thomas and Nielsen, 2005; Didelot and Maiden, 2010). The recombination rate varies greatly between different bacterial species, and high levels of recombination have been detected in *V. parahaemolyticus* (Cui et al., 2015). Of note, homologous recombination is of great significance for the survival and evolution of bacterial populations. Relative to spontaneous mutations, homologous recombination is associated with faster mutation speed than spontaneous mutation, which is important for a quick increase of the genetic diversity and the environmental adaptability potential of a population (Vos, 2009). Newly acquired mutated loci affect gene function or regulation, leading to new phenotypes of the corresponding strains, such as evading host immune response, developing multi-drug resistance, and increasing pathogenicity (Hacker and Carniel, 2001; Arnold et al., 2008). Interestingly, the MLST data generated in this study indicated that recombination replacement contributed 8.7 times more than point mutations to the genetic variations among all isolates. Therefore, recombination has a much greater effect than mutation on the generation and maintenance of genetic diversity in *V. parahaemolyticus*. In fact, the frequency of recombination and mutation in the clinical isolates was almost the same. On the one hand, that may be because the maintenance of some virulence-related functions requires gene stability and hence, the frequency of recombination is reduced. On the other hand, mutations can make the gene change at the level of function or regulation, leading to new phenotypes in corresponding strains, such as the virulence is enhanced, so the mutation frequency in clinical strains may increase to increase the virulence. In clinical strains, the frequency of recombination decreases and that of mutation increases, and ultimately, the two become close. However, the frequency of recombination in the food isolates was approximately 14 times that of mutation, indicating that recombination plays a greater role in maintaining genetic diversity of these isolates. Food isolates were obtained from different types of food sampled at different places and inhabit complex and diverse environments. Recombination might occur more frequently than mutation in these isolates to allow better adaptation to these environments.

Clonal, epidemic, and panmictic are three models of population structure that are used to distinguish bacterial pathogens. Clonal population contains strains derived from a common ancestor that diversified predominantly by mutation rather than recombination within linkages (Smith et al., 1993). In epidemic population, well-adapted clones become widely distributed, with recombination occurring much more frequently than mutation (Smith et al., 2000). Finally, in a panmictic population, all strains are potential partners without genetic mating restrictions, recombination occurs freely, and polymorphic sites are all at linkage equilibrium (Suerbaum et al., 1998).

*V. parahaemolyticus* bacteria are widely distributed in estuarine, marine, and coastal environments, suggesting they are able to rapidly adapt to various environments. Consistently, high levels of genotypic diversity were observed for *V. parahaemolyticus* isolates in this study and numerous singletons were detected in the population snapshot. Further, the *I^*S*^_*A*_* values were significantly different from zero for all the analyzed strain sets, indicating a tendency of linkage disequilibrium between the alleles. Of note, in the clonal population, recombination does not occur freely, and there is no random distribution of alleles in general; however, recombination can occur within different subpopulations (Gonzalez-Escalona et al., 2008). Consequently, the data suggest that *V. parahaemolyticus* populations follow the epidemic model of clonal expansion (Chao et al., 2011; Yan et al., 2011).

In the current study, when the eBURST algorithm and the ML tree were used to assess phylogenetic relationships, the results obtained were different. These two analysis tools have different advantages. eBURST analysis can be used to quickly identify clonal complexes and their ancestral STs, while the ML trees are better at revealing evolutionary relationships among different STs. The high degree of allelic diversity among the analyzed strains decreased the ability of goeBURST to identify related genotypes; evolutionary relationships are reliable only for identical or closely related strains (SLVs to TLVs) (Francisco et al., 2009). Therefore, simultaneous application of the two models would lead to more reliable conclusions than using either model alone.

Phylogenetic analysis revealed that the clinical isolates were more concentrated in the ML tree, while the food isolates were more dispersed, and few food isolates and clinical isolates were in the same cluster. A possible explanation is that the maintenance of some virulence-related functions of the clinical isolates requires gene stability, and the frequency of recombination is relatively reduced, while the food isolates frequently recombine to adapt to various environments. High-frequency recombination disrupts the vertical evolutionary relationship. The phylogeny is presented as a radial structure, which cannot reflect the sequence of evolution and the distance between species (Cui et al., 2015). Food isolates in the same cluster as clinical isolates may be potentially pathogenic strains that have contaminated the food but did not cause infection, resulting in outbreaks of foodborne diseases. Therefore, these strains should become key strains for the assessment of *V. parahaemolyticus* contamination. In addition, no ST3 and STs belonging to the pandemic CC3 were found in the food isolates, but ST3 was the most prevalent ST in the clinical isolates, if ST3 or STs belonging to CC3 is present in food isolates, these bacteria will be a priority for monitoring This observation can provide a new reference strategy for tracing the sources of clinical isolates and preventing the outbreaks of *V. parahaemolyticus* infection.

## Conclusion

The study revealed high genetic diversity of the analyzed *V. parahaemolyticus* strains, with many novel alleles and STs identified. Importantly, in both, food and clinical isolates, the housekeeping genes were mainly affected by purifying selection during the evolutionary process. We also showed that recombination plays a much greater role than mutation in the generation of genetic heterogeneity, particularly in the food isolates. The *I^*S*^_*A*_* values were significantly different from zero for all the analyzed strain sets, indicating that the alleles are in linkage disequilibrium in both food and clinical isolates. The clinical isolates showed a concentrated distribution in the ML tree, while the food isolates were more distinct. Of note, CC415 was the most prevalent CC among the food isolates, while CC3 was the most prevalent CC among the clinical isolates. Collectively, these observations suggest that while the food and clinical isolates of *V. parahaemolyticus* from Guangdong Province are similar, they show different evolutionary trends. This may help prevent large-scale spread of highly virulent strains.

## Data Availability Statement

The original contributions presented in the study are included in the article/Supplementary Material, further inquiries can be directed to the corresponding author/s.

## Author Contributions

QW and YD conceived the study. TL and HZ designed the experiments. MH and FJ conducted the experiments. MH wrote the manuscript. MC, RP, and LX contributed to data analysis. JZ supervised the experiment. JW, SW, and QY reviewed and edited the manuscript. All authors agreed to accountable for the content of the work.

## Conflict of Interest

The authors declare that the research was conducted in the absence of any commercial or financial relationships that could be construed as a potential conflict of interest.

## Publisher’s Note

All claims expressed in this article are solely those of the authors and do not necessarily represent those of their affiliated organizations, or those of the publisher, the editors and the reviewers. Any product that may be evaluated in this article, or claim that may be made by its manufacturer, is not guaranteed or endorsed by the publisher.

## Footnotes

1
https://pubmlst.org/organisms/vibrio-parahaemolyticus/

2
http://www.phyloviz.net
