# Supplementary material for: Genetic Diversity and Population Structure of Vibrio parahaemolyticus Isolated From Clinical and Food Sources
Source: Front Microbiol. 2021 Jul 27;12:708795. doi: 10.3389/fmicb.2021.708795 (PMC8353399; doi:10.3389/fmicb.2021.708795)
Supplement: Supplementary file 9 [file Table_1.docx]

Supplementary Table S1. The 162 strains and the allelic profiles of them used in this study

| strain name | Location | Source | Host | Serogroup | *dnaE* | *gyrB* | *recA* | *dtdS* | *pntA* | *pyrC* | *tnaA* | ST | CC |
| --- | --- | --- | --- | --- | --- | --- | --- | --- | --- | --- | --- | --- | --- |
| Vp1 | Conghua District | Aquatic product | Fish belly | O2 | 2 | 113 | 72 | 94 | 26 | 83 | 23 | 411 | Singleton |
| Vp25 | Zengcheng District | Aquatic product | Shrimp | O2 | 42 | 134 | 99 | 79 | 26 | 41 | 51 | 415 | 415 |
| Vp28 | Conghua District | Aquatic product | Shrimp | O1 | 137 | 238 | 3 | 194 | 122 | 11 | 131 | 445 | Singleton |
| Vp30 | Yuexiu District | Aquatic product | Shrimp | O1 | 111 | 5 | 22 | 34 | 20 | 171 | 24 | 1525 | Singleton |
| Vp32 | Yuexiu District | Aquatic product | Frozen Shrimp | O11 | 303 | 25 | 306 | 151 | 31 | 252 | 73 | 1288 | Singleton |
| Vp36 | Yuexiu District | Aquatic product | Shrimp | O5 | 28 | 144 | 3 | 138 | 26 | 177 | 61 | 2137 | Singleton |
| Vp38 | Panyu District | Aquatic product | Dried shrimp | O2 | 42 | 134 | 99 | 79 | 26 | 41 | 51 | 415 | 415 |
| Vp218 | Panyu District | Aquatic product | Shrimp | O4 | 47 | 91 | 166 | 471 | 79 | 45 | 121 | 2034 | Singleton |
| Vp223 | Conghua District | Aquatic product | Squid | O2 | 295 | 104 | 3 | 470 | 28 | 37 | 51 | 2035 | 1236 |
| Vp224 | Conghua District | Aquatic product | Fish belly | O8 | 42 | 134 | 99 | 79 | 26 | 41 | 51 | 415 | 415 |
| Vp227 | Conghua District | Aquatic product | Shrimp | O8 | 337 | 106 | 199 | 331 | 2 | 3 | 20 | 2165 | Singleton |
| Vp228 | Conghua District | Aquatic product | Yellow croaker | O2 | 28 | 39 | 230 | 19 | 61 | 195 | 1 | 888 | Singleton |
| Vp229 | Panyu District | Aquatic product | Shrimp | O2 | 28 | 164 | 310 | 378 | 23 | 82 | 57 | 1270 | Singleton |
| Vp230 | Zengcheng District | Aquatic product | Shrimp | O1 | 175 | 530 | 168 | 169 | 130 | 17 | 73 | 2126 | 2126 |
| Vp231 | Zengcheng District | Aquatic product | Frozen Shrimp | O1 | 42 | 134 | 99 | 79 | 26 | 41 | 51 | 415 | 415 |
| Vp232 | Conghua District | Aquatic product | Shrimp | O4 | 325 | 446 | 321 | 314 | 4 | 260 | 57 | 2039 | Singleton |
| Vp234 | Conghua District | Aquatic product | Shrimp | O12 | 93 | 343 | 144 | 19 | 23 | 201 | 23 | 2127 | Singleton |
| Vp236 | Conghua District | Aquatic product | Shrimp | O1 | 295 | 104 | 3 | 471 | 28 | 37 | 51 | 2128 | 1236 |
| Vp238 | Zengcheng District | Aquatic product | Shrimp | O11 | 42 | 134 | 99 | 79 | 26 | 37 | 51 | 2129 | 415 |
| Vp239 | Conghua District | Aquatic product | Shrimp | O1 | 295 | 104 | 3 | 471 | 28 | 37 | 51 | 2128 | 1236 |
| Vp240 | Zengcheng District | Aquatic product | Shrimp | O2 | 184 | 251 | 334 | 29 | 54 | 96 | 26 | 1564 | Singleton |
| Vp241 | Zengcheng District | Aquatic product | Shrimp | O2 | 9 | 21 | 15 | 13 | 4 | 10 | 26 | 12 | Singleton |
| Vp242  Vp254 | Zengcheng District  Zengcheng District | Aquatic product  Aquatic product | Shrimp  Shrimp | O4  O10 | 175  175 | 22  530 | 168  168 | 201  169 | 130  80 | 17  17 | 73  73 | 471  2412 | 471  2126 |
| strain name | Location | Source | Host | Serogroup | *dnaE* | *gyrB* | *recA* | *dtdS* | *pntA* | *pyrC* | *tnaA* | ST | CC |
| Vp257 | Zengcheng District | Aquatic product | Shrimp | O11 | 9 | 21 | 15 | 13 | 4 | 10 | 26 | 12 | Singleton |
| Vp258 | Panyu District | Aquatic product | Shrimp | O5 | 49 | 169 | 64 | 76 | 92 | 404 | 98 | 2130 | Singleton |
| Vp259 | Panyu District | Aquatic product | Shrimp | O2 | 60 | 147 | 339 | 133 | 26 | 3 | 252 | 2131 | Singleton |
| Vp264 | Panyu District | Aquatic product | Shrimp | O3 | 3 | 230 | 61 | 268 | 2 | 46 | 2 | 693 | Singleton |
| Vp266 | Panyu District | Aquatic product | Shrimp | O3 | 196 | 84 | 193 | 41 | 128 | 203 | 24 | 1498 | 1498 |
| Vp271 | Panyu District | Aquatic product | Shrimp | O12 | 11 | 75 | 64 | 67 | 124 | 7 | 50 | 452 | Singleton |
| Vp272 | Zengcheng District | Aquatic product | Shrimp | O3 | 42 | 134 | 99 | 79 | 26 | 41 | 51 | 415 | 415 |
| Vp273 | Panyu District | Aquatic product | Shrimp | O2 | 337 | 106 | 199 | 331 | 2 | 3 | 20 | 2165 | Singleton |
| Vp278 | Panyu District | Aquatic product | Shrimp | O1 | 392 | 531 | 102 | 65 | 28 | 37 | 12 | 2132 | Singleton |
| Vp279 | Panyu District | Aquatic product | Carp | O2 | 393 | 355 | 98 | 47 | 26 | 441 | 34 | 2413 | Singleton |
| Vp281 | Panyu District | Aquatic product | Cabezon | O1 | 11 | 75 | 64 | 67 | 124 | 7 | 50 | 452 | Singleton |
| Vp284 | Panyu District | Aquatic product | Carp | O2 | 394 | 52 | 15 | 47 | 23 | 14 | 276 | 2133 | Singleton |
| Vp286 | Panyu District | Aquatic product | Carp | O1 | 394 | 52 | 15 | 47 | 23 | 14 | 276 | 2133 | Singleton |
| Vp287 | Panyu District | Aquatic product | Carp | O1 | 28 | 164 | 310 | 378 | 23 | 82 | 57 | 1270 | Singleton |
| Vp292 | Zengcheng District | Aquatic product | Shrimp | O2 | 9 | 21 | 15 | 13 | 4 | 10 | 26 | 12 | Singleton |
| Vp293 | Panyu District | Aquatic product | Shrimp | O2 | 5 | 532 | 251 | 129 | 120 | 5 | 203 | 2134 | Singleton |
| Vp297 | Yuexiu District | Aquatic product | Prawn | O2 | 44 | 260 | 339 | 214 | 26 | 200 | 99 | 2135 | 515 |
| Vp299 | Liwan District | Aquatic product | Grass carp | O12 | 42 | 134 | 99 | 79 | 26 | 41 | 51 | 415 | 415 |
| Vp301 | Yuexiu District | Aquatic product | Squid | O2 | 42 | 134 | 15 | 79 | 26 | 41 | 51 | 2136 | 415 |
| Vp305 | Yuexiu District | Aquatic product | Shrimp | O8 | 44 | 58 | 257 | 371 | 4 | 3 | 238 | 1249 | Singleton |
| Vp306 | Panyu District | Aquatic product | Tilapia | O2 | 28 | 144 | 3 | 138 | 26 | 177 | 61 | 2137 | Singleton |
| Vp409 | Yuexiu District | Aquatic product | Grass carp | O1 | 198 | 409 | 35 | 356 | 26 | 92 | 186 | 1124 | Singleton |
| Vp410 | Yuexiu District | Aquatic product | Prawn | O2 | 49 | 169 | 67 | 76 | 92 | 127 | 98 | 1820 | Singleton |
| Vp412 | Yuexiu District | Aquatic product | Frozen Shrimp | O2 | 3 | 230 | 61 | 268 | 2 | 46 | 2 | 693 | Singleton |
| Vp413 | Yuexiu District | Aquatic product | Shrimp | O2 | 42 | 134 | 99 | 79 | 26 | 41 | 51 | 415 | 415 |
| strain name | Location | Source | Host | Serogroup | *dnaE* | *gyrB* | *recA* | *dtdS* | *pntA* | *pyrC* | *tnaA* | ST | CC |
| Vp416 | Panyu District | Aquatic product | Frozen Shrimp | O2 | 395 | 5 | 391 | 150 | 47 | 3 | 26 | 2138 | Singleton |
| Vp418 | Yuexiu District | Aquatic product | Prawn | O2 | 148 | 355 | 74 | 19 | 127 | 101 | 202 | 847 | Singleton |
| Vp419 | Panyu District | Aquatic product | Shrimp | O2 | 5 | 25 | 103 | 480 | 149 | 418 | 169 | 2139 | Singleton |
| Vp420 | Panyu District | Aquatic product | Shrimp | O2 | 394 | 52 | 15 | 47 | 23 | 14 | 276 | 2133 | Singleton |
| Vp421 | Yuexiu District | Aquatic product | Frozen Shrimp | O2 | 42 | 134 | 99 | 79 | 26 | 41 | 238 | 415 | 415 |
| Vp422 | Yuexiu District | Aquatic product | Shrimp | O11 | 111 | 5 | 22 | 34 | 20 | 171 | 24 | 1525 | Singleton |
| Vp423 | Yuexiu District | Aquatic product | Shrimp | O2 | 55 | 15 | 31 | 55 | 18 | 58 | 46 | 114 | 114 |
| Vp424 | Yuexiu District | Aquatic product | Shrimp | O2 | 28 | 144 | 3 | 138 | 26 | 177 | 61 | 2137 | Singleton |
| Vp425 | Yuexiu District | Aquatic product | Frozen Shrimp | O2 | 42 | 134 | 210 | 79 | 26 | 41 | 51 | 2140 | 415 |
| Vp426 | Zhanjiang | Ready-to-eat food | Chencun rice noodles | O1 | 126 | 427 | 123 | 103 | 103 | 7 | 26 | 2141 | Singleton |
| Vp427 | Zengcheng District | Aquatic product | Pacific Saury | O1 | 174 | 229 | 99 | 198 | 18 | 176 | 130 | 2142 | Singleton |
| Vp428 | Panyu District | Aquatic product | Squid | O12 | 3 | 230 | 61 | 268 | 2 | 46 | 2 | 693 | Singleton |
| Vp429 | Zengcheng District | Aquatic product | Shrimp | O3 | 42 | 1 | 62 | 241 | 26 | 85 | 20 | 1583 | Singleton |
| Vp430 | Shenzhen | Aquatic product | Shrimp | O2 | 111 | 404 | 25 | 149 | 115 | 164 | 118 | 2143 | Singleton |
| Vp431 | Panyu District | Aquatic product | Shrimp | O3 | 35 | 413 | 164 | 360 | 18 | 45 | 9 | 2144 | Singleton |
| Vp432 | Panyu District | Aquatic product | Squid | O3 | 400 | 122 | 31 | 481 | 47 | 11 | 17 | 2145 | Singleton |
| Vp433 | Zengcheng District | Ready-to-eat food | Stewed pork | O12 | 303 | 25 | 306 | 151 | 31 | 252 | 73 | 1288 | Singleton |
| Vp435 | Shenzhen | Aquatic product | Shrimp | O2 | 31 | 106 | 135 | 74 | 26 | 212 | 54 | 657 | 564 |
| Vp436 | Conghua District | Ready-to-eat food | Pickled chicken feet | O2 | 71 | 345 | 246 | 167 | 177 | 69 | 47 | 815 | Singleton |
| Vp437 | Conghua District | Aquatic product | Shrimp | O10 | 325 | 446 | 321 | 314 | 4 | 280 | 57 | 2039 | Singleton |
| Vp438 | Shenzhen | Aquatic product | Squid | O1 | 66 | 90 | 31 | 72 | 21 | 94 | 32 | 2146 | Singleton |
| Vp439 | Shenzhen | Aquatic product | Pacific Saury | O3 | 60 | 354 | 250 | 305 | 26 | 54 | 84 | 846 | 846 |
| Vp440 | Zhanjiang | Aquatic product | Squid | O4 | 31 | 106 | 135 | 74 | 37 | 212 | 54 | 564 | 564 |
| strain name | Location | Source | Host | Serogroup | *dnaE* | *gyrB* | *recA* | *dtdS* | *pntA* | *pyrC* | *tnaA* | ST | CC |
| Vp441 | Shenzhen | Aquatic product | Shrimp | O10 | 10 | 1 | 62 | 95 | 50 | 85 | 2 | 1825 | Singleton |
| Vp442 | Shenzhen | Aquatic product | Shrimp | O2 | 19 | 86 | 109 | 433 | 46 | 68 | 57 | 1838 | Singleton |
| Vp443 | Shenzhen | Aquatic product | Squid | O2 | 60 | 354 | 109 | 305 | 26 | 54 | 84 | 2149 | 846 |
| Vp445 | Shenzhen | Aquatic product | Shrimp | O1 | 215 | 344 | 144 | 76 | 48 | 232 | 26 | 809 | Singleton |
| Vp446 | Panyu District | Aquatic product | Shrimp | O5 | 394 | 52 | 15 | 47 | 23 | 14 | 276 | 2133 | Singleton |
| Vp447 | Panyu District | Aquatic product | Frozen Shrimp | O12 | 28 | 164 | 310 | 378 | 23 | 82 | 57 | 1270 | Singleton |
| Vp448 | Zhanjiang | Ready-to-eat food | Stewed pork | O5 | 11 | 106 | 192 | 220 | 71 | 73 | 17 | 1352 | Singleton |
| Vp449 | Heyuan | Ready-to-eat food | Shredded Chicken | O1 | 51 | 4 | 77 | 67 | 213 | 8 | 24 | 1228 | Singleton |
| Vp450 | Shenzhen | Aquatic product | Shrimp | O2 | 148 | 355 | 74 | 19 | 127 | 101 | 202 | 847 | Singleton |
| Vp451 | Yuexiu District | Aquatic product | Prawn | O1 | 3 | 403 | 392 | 353 | 182 | 11 | 66 | 2150 | Singleton |
| Vp453 | Conghua District | Aquatic product | Grass carp | O8 | 44 | 260 | 3 | 214 | 26 | 200 | 99 | 515 | 515 |
| Vp455 | Shaoguan | Aquatic product | Shrimp | O2 | 93 | 77 | 74 | 19 | 23 | 201 | 23 | 532 | Singleton |
| Vp456 | Zhanjiang | Aquatic product | Tilapia | O10 | 396 | 419 | 226 | 482 | 142 | 11 | 26 | 2156 | Singleton |
| Vp457 | Zhanjiang | Aquatic product | Shrimp | O2 | 397 | 195 | 123 | 66 | 46 | 7 | 26 | 2157 | Singleton |
| Vp458 | Heyuan | Aquatic product | Shrimp | O2 | 148 | 355 | 74 | 19 | 127 | 101 | 202 | 847 | Singleton |
| Vp459 | Zhanjiang | Aquatic product | Shrimp | O10 | 398 | 44 | 393 | 435 | 26 | 39 | 73 | 2158 | Singleton |
| Vp460 | Shenzhen | Aquatic product | Frozen Shrimp | O1 | 35 | 279 | 25 | 317 | 26 | 323 | 26 | 2159 | Singleton |
| Vp461 | Heyuan | Aquatic product | Shrimp | O2 | 148 | 355 | 74 | 19 | 127 | 101 | 202 | 847 | Singleton |
| Vp465 | Shenzhen | Aquatic product | Frozen Shrimp | O3 | 4 | 13 | 11 | 38 | 18 | 9 | 61 | 2160 | Singleton |
| Vp466 | Shaoguan | Aquatic product | Carp | O4 | 42 | 535 | 89 | 13 | 30 | 307 | 50 | 2161 | Singleton |
| Vp467 | Zhanjiang | Aquatic product | Prawn | O6 | 399 | 536 | 61 | 183 | 179 | 216 | 26 | 2162 | Singleton |
| Vp468 | Zhanjiang | Aquatic product | Shrimp | O2 | 3 | 106 | 177 | 349 | 199 | 43 | 9 | 2163 | Singleton |
| Vp469 | Shaoguan | Aquatic product | Carp | O2 | 394 | 52 | 15 | 47 | 23 | 14 | 276 | 2133 | Singleton |
| Vp470 | Conghua District | Ready-to-eat food | Cold dish | O5 | 9 | 21 | 15 | 13 | 4 | 10 | 26 | 12 | Singleton |
| Vp471 | Shaoguan | Aquatic product | Shrimp | O4 | 31 | 73 | 381 | 215 | 18 | 249 | 8 | 2164 | Singleton |
| strain name | Location | Source | Host | Serogroup | *dnaE* | *gyrB* | *recA* | *dtdS* | *pntA* | *pyrC* | *tnaA* | ST | CC |
| Vp475 | Panyu District | Aquatic product | Shrimp | O2 | 160 | 203 | 15 | 118 | 82 | 5 | 80 | 376 | Singleton |
| Vp477 | Liwan District | Aquatic product | Yellow catfish | O1 | 10 | 1 | 62 | 95 | 50 | 85 | 2 | 1825 | Singleton |
| Vp478 | Zengcheng District | Aquatic product | Shrimp | O2 | 19 | 86 | 109 | 433 | 46 | 68 | 57 | 1838 | Singleton |
| Vp479 | Shaoguan | Aquatic product | Shrimp | O10 | 337 | 106 | 199 | 331 | 2 | 3 | 20 | 2165 | Singleton |
| Vp481 | Zhanjiang | Aquatic product | Squid | O4 | 118 | 253 | 72 | 76 | 50 | 184 | 54 | 490 | Singleton |
| Vp482 | Conghua District | Aquatic product | Stewed pork | O4 | 295 | 104 | 3 | 471 | 28 | 37 | 51 | 2128 | 1236 |
| Vp483 | Shaoguan | Aquatic product | Carp | O12 | 391 | 7 | 31 | 13 | 43 | 440 | 26 | 2166 | Singleton |
| Vp484 | Shaoguan | Aquatic product | Tilapia | O8 | 149 | 184 | 31 | 76 | 98 | 11 | 84 | 338 | Singleton |
| Vp486 | Shantou City | Aquatic product | Aconitum fish | O10 | 35 | 138 | 101 | 167 | 80 | 3 | 47 | 2167 | Singleton |
| Vp487 | Zhanjiang | Aquatic product | Carp | O10 | 11 | 106 | 192 | 220 | 71 | 73 | 17 | 1352 | Singleton |
| Vp488 | Panyu District | Aquatic product | Tilapia | O1 | 35 | 278 | 75 | 246 | 73 | 116 | 157 | 2168 | Singleton |
| Vp489 | Yuexiu District | Aquatic product | Shrimp | O1 | 331 | 528 | 390 | 191 | 165 | 95 | 210 | 2169 | Singleton |
| Vp490 | Zengcheng District | Aquatic product | Shrimp | O1 | 93 | 77 | 144 | 19 | 23 | 201 | 23 | 532 | Singleton |
| Vp491 | Liwan District | Aquatic product | Carp | O1 | 55 | 15 | 246 | 55 | 18 | 58 | 275 | 2170 | Singleton |
| Vp492 | Shaoguan | Aquatic product | Squid | O4 | 89 | 138 | 15 | 89 | 57 | 11 | 57 | 2171 | Singleton |
| Vp493 | Shenzhen | Aquatic product | Frozen Shrimp | O10 | 4 | 13 | 11 | 38 | 28 | 9 | 207 | 2172 | Singleton |
| Vp495 | Shenzhen | Aquatic product | Squid | O2 | 98 | 529 | 136 | 107 | 77 | 11 | 62 | 2173 | Singleton |
| Vp496 | Zengcheng District | Aquatic product | Shrimp | O11 | 93 | 224 | 75 | 139 | 117 | 223 | 124 | 919 | Singleton |
| Vp497 | Heyuan | Aquatic product | Shrimp | O2 | 175 | 49 | 168 | 201 | 130 | 17 | 73 | 2174 | 471 |
| Vp498 | Shenzhen | Aquatic product | Frozen Shrimp | O2 | 133 | 67 | 4 | 79 | 43 | 63 | 23 | 281 | Singleton |
| Vp499 | Heyuan | Aquatic product | Shrimp | O5 | 55 | 15 | 31 | 55 | 18 | 3 | 46 | 2175 | 114 |
| Vp500 | Shaoguan | Aquatic product | Shrimp | O2 | 148 | 355 | 74 | 19 | 127 | 101 | 202 | 847 | Singleton |
| Vp501 | Shenzhen | Aquatic product | Carp | O10 | 106 | 202 | 154 | 27 | 235 | 99 | 237 | 2176 | Singleton |
| SZ28 | Shenzhen | Human | Anal swab | O1 | 3 | 533 | 19 | 416 | 60 | 307 | 33 | 2147 | Singleton |
| SZ29 | Shenzhen | Human | Anal swab | O1 | 112 | 4 | 77 | 92 | 60 | 8 | 26 | 1823 | Singleton |
| strain name | Location | Source | Host | Serogroup | *dnaE* | *gyrB* | *recA* | *dtdS* | *pntA* | *pyrC* | *tnaA* | ST | CC |
| SZ30 | Shenzhen | Human | Anal swab | O3 | 3 | 4 | 19 | 4 | 29 | 4 | 22 | 3 | 3 |
| SZ31 | Shenzhen | Human | Anal swab | O3 | 3 | 4 | 19 | 4 | 29 | 4 | 22 | 3 | 3 |
| SZ32 | Shenzhen | Human | Anal swab | O3 | 3 | 4 | 19 | 4 | 29 | 4 | 22 | 3 | 3 |
| SZ33 | Shenzhen | Human | Anal swab | O3 | 3 | 4 | 19 | 4 | 29 | 4 | 22 | 3 | 3 |
| SZ34 | Shenzhen | Human | Anal swab | O3 | 3 | 4 | 19 | 4 | 29 | 4 | 22 | 3 | 3 |
| SZ35 | Shenzhen | Human | Anal swab | O3 | 3 | 4 | 19 | 4 | 29 | 4 | 22 | 3 | 3 |
| SZ36 | Shenzhen | Human | Anal swab | O3 | 150 | 29 | 31 | 95 | 269 | 8 | 277 | 2148 | Singleton |
| SZ37 | Shenzhen | Human | Anal swab | O1 | 28 | 4 | 82 | 88 | 63 | 69 | 1 | 8 | 8 |
| SZ38 | Shenzhen | Human | Anal swab | O11 | 3 | 4 | 19 | 4 | 29 | 4 | 22 | 3 | 3 |
| SZ39 | Shenzhen | Human | Anal swab | O1 | 28 | 4 | 82 | 88 | 63 | 69 | 22 | 2151 | 8 |
| SZ40 | Shenzhen | Human | Anal swab | O1 | 3 | 4 | 19 | 4 | 29 | 4 | 22 | 3 | 3 |
| SZ42 | Shenzhen | Human | Anal swab | O2 | 31 | 131 | 135 | 91 | 19 | 221 | 26 | 2152 | 587 |
| SZ43 | Shenzhen | Human | Anal swab | O4 | 70 | 175 | 19 | 81 | 49 | 74 | 62 | 2153 | Singleton |
| SZ44 | Shenzhen | Human | Anal swab | O2 | 31 | 131 | 135 | 91 | 19 | 221 | 26 | 2152 | 587 |
| SZ45 | Shenzhen | Human | Anal swab | O4 | 112 | 534 | 93 | 163 | 6 | 104 | 23 | 2154 | Singleton |
| SZ47 | Shenzhen | Human | Anal swab | O3 | 3 | 4 | 19 | 4 | 29 | 4 | 22 | 3 | 3 |
| SZ48 | Shenzhen | Human | Anal swab | O1 | 19 | 74 | 61 | 68 | 48 | 11 | 22 | 150 | Singleton |
| SZ50 | Shenzhen | Human | Anal swab | O3 | 3 | 4 | 19 | 4 | 29 | 4 | 22 | 3 | 3 |
| SZ51 | Shenzhen | Human | Anal swab | O2 | 31 | 131 | 135 | 91 | 19 | 221 | 26 | 2152 | 587 |
| SZ52 | Shenzhen | Human | Anal swab | O3 | 3 | 4 | 192 | 4 | 29 | 4 | 22 | 2155 | 3 |
| SZ53 | Shenzhen | Human | Anal swab | O11 | 31 | 131 | 135 | 91 | 19 | 221 | 26 | 2152 | 587 |
| SZ54 | Shenzhen | Human | Anal swab | O11 | 31 | 131 | 135 | 91 | 19 | 221 | 26 | 2152 | 587 |
| SZ57 | Shenzhen | Human | Anal swab | O3 | 3 | 4 | 19 | 4 | 29 | 4 | 22 | 3 | 3 |
| SZ58 | Shenzhen | Human | Anal swab | O2 | 49 | 169 | 67 | 76 | 92 | 127 | 98 | 1820 | Singleton |
| L1 | Guangzhou | Human |  |  | 3 | 4 | 19 | 4 | 29 | 4 | 22 | 3 | 3 |
| strain name | Location | Source | Host | Serogroup | *dnaE* | *gyrB* | *recA* | *dtdS* | *pntA* | *pyrC* | *tnaA* | ST | CC |
| L2 | Guangzhou | Human |  |  | 3 | 4 | 19 | 4 | 29 | 4 | 22 | 3 | 3 |
| L3 | Guangzhou | Human |  |  | 3 | 4 | 19 | 4 | 29 | 4 | 22 | 3 | 3 |
| L5 | Guangzhou | Human |  |  | 3 | 4 | 19 | 4 | 29 | 4 | 22 | 3 | 3 |
| L6 | Guangzhou | Human |  |  | 3 | 4 | 19 | 4 | 29 | 4 | 22 | 3 | 3 |
| L7 | Guangzhou | Human |  |  | 3 | 4 | 19 | 4 | 29 | 4 | 22 | 3 | 3 |
| L8 | Guangzhou | Human |  |  | 3 | 4 | 19 | 4 | 29 | 4 | 22 | 3 | 3 |
| r16 | Guangzhou | Human |  |  | 196 | 84 | 193 | 41 | 128 | 203 | 230 | 2358 | 1498 |
| r46 | Guangzhou | Human |  |  | 3 | 4 | 19 | 4 | 29 | 4 | 22 | 3 | 3 |
| r75 | Guangzhou | Human |  |  | 3 | 4 | 19 | 4 | 29 | 4 | 22 | 3 | 3 |
| r79-2 | Guangzhou | Human |  |  | 3 | 4 | 19 | 4 | 29 | 4 | 22 | 3 | 3 |
| VpL61 | Shenzhen | Human |  | O3 | 3 | 4 | 19 | 4 | 29 | 4 | 22 | 3 | 3 |
| VpL65 | Shenzhen | Human |  | O3 | 3 | 4 | 19 | 4 | 29 | 4 | 22 | 3 | 3 |
| VpL71 | Shenzhen | Human |  | O1 | 28 | 4 | 82 | 88 | 63 | 69 | 1 | 8 | 8 |
| VpL83 | Shenzhen | Human |  | O2 | 31 | 131 | 1 | 91 | 19 | 221 | 26 | 2359 | 587 |
| VpL88 | Shenzhen | Human |  | O2 | 202 | 279 | 208 | 242 | 4 | 54 | 57 | 2360 | Singleton |
