# Supplementary material for: Genetic Diversity and Population Structure of Vibrio parahaemolyticus Isolated From Clinical and Food Sources
Source: Front Microbiol. 2021 Jul 27;12:708795. doi: 10.3389/fmicb.2021.708795 (PMC8353399; doi:10.3389/fmicb.2021.708795)
Supplement: Supplementary file 10 [file Table_2.docx]

Supplementary Table S2. Genes and primers used for MLST

| Gene name | Primers | | Product |
| --- | --- | --- | --- |
| *recA* | *recA*-1F | tgtaaaacgacggccagtGAAACCATTTCAACGGGTTC | RecA protein |
|  | *recA*-1R | caggaaacagctatgaccCCATTGTAGCTGTACCAAGCACCC |  |
| *gyrB* | *gyrB*-1F | tgtaaaacgacggccagtGAAGGBGGTATTCAAGC | DNA gyrase, subunit B |
|  | *gyrB*-1R | caggaaacagctatgaccGAGTCACCCTCCACWATGTA |  |
| *dnaE* | *dnaE*-1F | tgtaaaacgacggccagtCGRATMACCGCTTTCGCCG | DNA polymerase III, alpha subunit |
|  | *dnaE*-1R | caggaaacagctatgaccGAKATGTGTGAGCTGTTTGC |  |
| *dtdS* | *dtdS*-1F | tgtaaaacgacggccagtTGGCCATAACGACATTCTGA | Threonine dehyrogenase |
|  | *dtdS*-1R | caggaaacagctatgaccGAGCACCAACGTGTTTAGC |  |
| *pntA* | *pntA*-1F | tgtaaaacgacggccagtACGGCTACGCAAAAGAAATG | Transhydrogenase alpha subunit |
|  | *pntA*-1R | caggaaacagctatgaccTTGAGGCTGAGCCGATACTT |  |
| *pyrC* | *pyrC*-1F | tgtaaaacgacggccagtAGCAACCGGTAAAATTGTCG | Dihydroorotase |
|  | *pyrC*-1R | caggaaacagctatgaccCAGTGTAAGAACCGGCACAA |  |
| *tnaA* | *tnaA*-1F | tgtaaaacgacggccagtTGTACGAAATTGCCACCAAA | Tryptophanase |
|  | *tnaA*-1R | caggaaacagctatgaccAATATTTTCGCCGCATCAAC |  |
